# Ginsenoside F1 Ameliorates Endothelial Cell Inflammatory Injury and Prevents Atherosclerosis in Mice through A20-Mediated Suppression of NF-kB Signaling

**DOI:** 10.3389/fphar.2017.00953

**Published:** 2017-12-22

**Authors:** Meng Qin, Yun Luo, Shan Lu, Jing Sun, Ke Yang, Guibo Sun, Xiaobo Sun

**Affiliations:** ^1^Institute of Medicinal Plant Development, Peking Union Medical College, Chinese Academy of Medical Sciences, Beijing, China; ^2^Beijing Key Laboratory of Innovative Drug Discovery of Traditional Chinese Medicine (Natural Medicine) and Translational Medicine, Peking Union Medical College, Chinese Academy of Medical Sciences, Beijing, China; ^3^Key Laboratory of Bioactive Substances and Resource Utilization of Chinese Herbal Medicine, Ministry of Education, Beijing, China; ^4^Key Laboratory of Efficacy Evaluation of Chinese Medicine against Glyeolipid Metabolism Disorder Disease, State Administration of Traditional Chinese Medicine, Beijing, China; ^5^Institute of Chinese Materia Medica, China Academy of Chinese Medical Sciences, Beijing, China; ^6^Collaborative Innovation Center of Yangtze River Delta Region Green Pharmaceuticals, Zhejiang University of Technology, Hangzhou, China

**Keywords:** Ginsenoside F1, ox-LDL, HUVECs, inflammatory injury, A20, atherosclerosis

## Abstract

Atherosclerosis (AS) is a chronic inflammatory disease and endothelial cell injury is the initial event. In this study, we investigated the protective effects of ginsenoside F1 (GF1) on AS and the potential molecular mechanisms of ox-LDL induced endothelial injury. ApoE-/- mice were fed a high fat diet and orally treated with GF1 (50 mg/kg/day) for 8 weeks. Atherosclerotic plaque and LOX-1, TLR4, NF-κB expression levels in the aortic root and inflammatory factor MPO in whole body were measured. The treatment with GF1 induced a remarkable reduction in the atherosclerotic lesion area, LOX-1, TLR4 expression and decreased the MPO distribution. Meanwhile, *in vitro* study, we confirmed that GF1 treatment greatly increased ox-LDL-injured endothelial cell viability, ameliorated LOX-1, TLR4 expression levels and reduced monocytes adhesion. Protein microarray demonstrated that GF1 significantly inhibited G-CSF, ICAM-1, MIP-1δ, IL-1α, IL-15, IL-16 levels. Mechanistically, the GF1 treatment suppressed the NF-κB nuclear translocation. Furthermore, our data indicated that GF1 significantly increased A20 expression level and A20 siRNA markedly abolished the attenuation of GF1 on NF-κB nuclear translocation and inflammatory factors expression. Our results suggest that the GF1 may be a potential drug for anti-atherosclerosis.

## Introduction

Atherosclerosis (AS) is a chronically inflammatory disease in the medium- and large-sized arteries, mainly involving interactions in endothelial cells, macrophages, vascular smooth muscle cells, and cytokines (Ross, 1993). Oxidized low density lipoprotein (ox-LDL) induced endothelial cell injury is the initial event in the development of AS (Glass and Witztum, 2001). Ox-LDL-damaged endothelial cells release adhesion molecules, which causes monocytes migration and differentiation into macrophages. Then, ox-LDL stimulates transformation of macrophages and vascular smooth muscle cells into lipid-rich foam cells, promoting AS. It has been demonstrated that ox-LDL binding its receptors lectin-like receptor for oxidized (ox)-LDL (LOX-1) could activate inflammatory response through multiple signal transduction pathways (Morawietz, 2007). Therefore, it is important for AS treatment by reducing endothelial cell injury and inflammatory response.

Zinc finger protein A20 (tumor necrosis factor alpha-induced protein 3), a potent negative feedback inhibitor of the NF-κB signaling, has been widely reported in auto-inflammatory disease (Zilberman-Rudenko et al., 2016), stroke (Zhan et al., 2016), cancer (Chen et al., 2015), and AS (Wolfrum et al., 2007). It has been demonstrated that A20 overexpression protected endothelial cells apoptosis through inhibiting CD40-CD40L-mediated NF-κB signaling (Longo et al., 2003). Ox-LDL activates NADPH through LOX-1, which generates lots of reactive oxygen species (ROS) (Tsai et al., 2011). Damaged endothelial cell loses its biological function, which accelerates inflammatory response and the plaque instability (Tuttolomondo et al., 2012). Meanwhile, LOX-1 induces NF-κB activation and upregulates inflammatory factor expression, which increases inflammatory responses. On the other hand, NF-κB activation modulates apoptosis genes, which could mediate endothelial cell apoptosis and promote AS development (Bao et al., 2013; Wang et al., 2013). In recent years, toll like receptors (TLRs) are involved in LOX-1 mediated cell injury. It has been demonstrated TLR4 is found in atherosclerotic plaque (Gargiulo et al., 2015). Thus, how to modulate TLR4, LOX-1, and A20 mediated NF-κB activation could be a valid target for AS treatment.

Ginsenosides, the main components in *Panax ginseng* C. A. Mey, exhibit strong anti-inflammation, anti-aging, and anti-oxidation activities (Lu et al., 2009; Shen et al., 2016; Wang C.Z. et al., 2016). Ginsenoside F1 (GF1) is a metabolite produced by hydrolysis of the ginsenosides Re and Rg1 (Kim et al., 2012). According to recent studies, GF1 exerts strong anti-aging, anti-oxidation, anticancer effects (Kim et al., 2015). Moreover, it is exciting that we could obtain abundant GF1 through ginsenoside Rg1 bioconversion, which promotes the study of GF1 (Kim et al., 2012; Wang Y. et al., 2016). However, there is few reports indicating the endothelial protection and anti-atherosclerosis effects of GF1.

In our current investigation, our goal was to explore the protective effects and molecular mechanisms of GF1 against ox-LDL-induced endothelial cell inflammatory injury. Meanwhile, we also investigated the anti-inflammatory effects of GF1 in high fat diet induced ApoE-/- atherosclerosis mice.

## Materials and Methods

### Ethics Statement

All experiments using mice were approved by the Institutional Animal Care and Use Committee (IACUC) at the Chinese Academy of Medical Sciences and Peking Union Medical College, Beijing, China (Luo et al., 2015a) and NIH Guidelines for the Care and Use of Laboratory Animals (McGrath et al., 2010).

### Materials

Ginsenoside F1 (purity > 98%) was purchased from Shanghai Winherb Medical Technology Company (China). Probucol was obtained from Qilu Pharmaceutical Co., Ltd. (Jinan, China). Ox-LDL (by copper ion-induced LDL oxidation, Malondialdehyde > 40 nmol/mg) was acquired from Union-Bio Technology (Beijing, China). Cell Counting Kit-8 (CCk-8) was gained from Dojindo (Kumamoto, Kyushu, Japan). Myeloperoxidase (MPO) fluorescent probe was purchased from Caliper Life Sciences, Inc. (Hopkinton, MA, United States). CM-dil was obtained from Amersco (Solon, OH, United States). Dimethylsulfoxide (DMSO), DAPI and oil red O were acquired from Sigma–Aldrich (St. Louis, MO, United States). All the antibodies were purchased from Abcam (Cambridge, England).

### Animals

Six-week-old (17 ± 1 g) male C57BL/6 mice and ApoE-/- mice with a C57BL/6 background were purchased from the Experimental Animal Center of Beijing University of Medical Sciences (Beijing, China) and maintained in a temperature-controlled facility (temperature: 22 ± 1°C; humidity: 60%) with a 14 h light/10 h dark cycle in conventional cages. Forty mice were randomly divided into four experimental groups (*n* = 10/group): (I) C57BL/6 N mice, the control group; (II) ApoE-/- mice group; (III) ApoE-/- mice + GF1 group; (IV) ApoE-/- mice + Probucol group. All mice were fed with a high fat diet (HFD, 0.3% cholesterol and 20% pork fat) for 8 weeks. GF1 (50 mg/kg/day, i.g.) and Probucol (2 g/kg, i.g.) were dissolved in carboxymethyl cellulose sodium (CMC-Na). Oral administration were given to mice every day for 8 weeks. The control and model groups received the aseptic 0.5% CMC-Na treatment every day (i.g., 0.1 ml/10g).

### *In Vivo* Myeloperoxidase (MPO) Detection

The MPO *in vivo* biodistribution was performed according to a previous study (Hong et al., 2016). Briefly, mice were given an intraperitoneal injection of probe at a dose of 150 mg/kg for 15 min. The MPO distribution was viewed using the IVIS Living Image^®^ 4.4 (Caliper Life Sciences, Hopkinton, MA, United States), which captured visible light photographs and luminescent images at 750 m excitation wavelength. The immunofluorescence revealed a high MPO expression, indicating that the inflammatory site *in vivo*. Living Image software (Version 4.2) was used for quantitative analysis. All the mice were imaged with identical instrument settings, and all the groups at each time point were under the same scale bar.

### Atherosclerotic Plaques Analysis

Serial sections of the aortic valve were stained with oil red O and *en face* of the proximal aorta was stained with Sudan IV as previously reported (Luo et al., 2015b). Briefly, obtained hearts were embedded with OCT, and serial 8-μm-thick frozen sections with 40-μm intervals were obtained and stained with oil red O. The images of plaque size and lesion area were analyzed using IPP 6.0 software (Media Cybernetics, Rockville, MD, United States).

### LOX-1 and TLR4 Expression Analysis

LOX-1 and TLR4 expression in aortic valve were detected by immunofluorescence as previously reported (Sun et al., 2013). Briefly, 8-μm-thick frozen sections were incubated at 4°C overnight with anti-LOX-1 and TLR4 rabbit polyclonal antibodies (1:50). Then, the sections were incubated with anti-rabbit FITC goat antibodies (1:100) for 1 h at room temperature and observed by an upright fluorescent microscopy. LOX-1 and TLR4 expression was quantified by IPP 6.0 software.

### Cell Culture and Treatment

Human umbilical vein endothelial cells (HUVECs) were isolated from fresh human umbilical veins and cultured on 1% gelatin-coated plastic dishes as previously described (Sun et al., 2013). The neonate cords were donated by the Maternal and Child Care Service Centre in Beijing, China. The study protocol was explained and all participating donors were given written informed consents. Briefly, the VascuLife Basal Medium with growth supplements, streptomycin (100 U/mL) and penicillin (100 U/mL) were used for the HUVECs culture. Passages 3–5 of the HUVECs from different donors were used for the experiments.

Ginsenoside F1 was dissolved in DMSO (100 μg/μl) and the cells were treated with diluted GF1 (1, 4, and 16 μM) for 24 h and then exposed to 70 μg/ml ox-LDL for an additional 24 h. Cell viability was detected using a cell counting kit-8 assay according to the manufacturer’s instruction.

Human monocyte leukemia cell line THP-1 was purchased from the Type Culture Collection of the Chinese Academy of Sciences (Shanghai, China), and cultured in RPMI-1640 medium supplemented with 10% (v/v) fetal bovine serum (FBS) and 100 U/ml penicillin-streptomycin. Cells were seeded in a humidified incubator at 37°C in a 5% CO_2_ atmosphere.

### Monocyte-HUVECs Adhesion Assay

For monocyte adhesion studies, HUVECs were cultured in 6-well plates, pretreatment with GF1 (16 μM) for 24 h, and then stimulated with 70 μg/mL ox-LDL for 24 h. THP-1 cells were incubated 1640 containing 10% FBS and 5 μM CM-dil at 37°C for 1 h to label the monocytes with fluorescence, and then washed three times with fresh medium 1640. After labeling the THP-1 cells with CM-dil, ox-LDL treated HUVECs were washed and a suspension of prepared THP-1 cells (1 × 10^6^ cells/mL) was added for 30 min. Non-adherent THP-1 cells were removed by washing and adherent cells were counted using a fluorescence microscope. The number of attached leukocytes was counted by the Image J software every 20 microscopic fields.

### Protein Microarray

The HUVECs were pretreated with GF1 (16 μM) 24 h and incubated with ox-LDL for another 24 h. The supernatant was obtained and concentrated. The protein concentration was measured by BCA assay. The protein microarray was determined by CapitalBio Corporation (Beijing, China) according to our previous study (Qin et al., 2015).

### Quantitative Real-Time PCR

The HUVECs were incubated with ox-LDL and pretreated with GF1 (16 μM) 24 h before the testing. Total RNA was extracted using TRIzol (Invitrogen, Carlsbad, CA, United States). About 2 μg of total RNA was reversely transcribed using the GoScript^TM^ Reverse Transcription System (Promega). cDNA was synthesized from the isolated RNA, and cycle time values were obtained using real-time RT-PCR with the Power SYBR Premix Ex Taq^TM^ II (TaKaRa Biotechnology, Dalian, China) in an iQ5 Real-time PCR detection system and analysis software (Bio-Rad, Santa Rosa, CA, United States) as previously described (Qin et al., 2015). The cycle number at which transcripts could be detected (CT) was normalized to the cycle number of GAPDH gene detection, and referred to as ΔCT. Primers (**Table 1**) were designed using premier primer Software 6.0 (Canadian Premier Life Insurance Company, ON, Canada).

**Table 1:** RT-PCR primers for analysis.

Gene	Direction	Sequence
GAPDH	Forward	5′-GTGGTCTCCTCTGACTTCAACA-3′
	Reverse	5′-CTCTTCCTCTTGTGCTCTTGCT-3′
LOX-1	Forward	5′-GACAGACAGACAGACAGACACA-3′
	Reverse	5′-CAGAGTTGAGAAGAGTTCATCTACA-3′
TLR4	Forward	5′-GTCTGGCTGGTTTAGAGTC-3′
	Reverse	5′-CACTCACCAGGGAAAATG -3′

### Western Blot Analysis

Human umbilical vein endothelial cells were harvested, washed with PBS, and lysed with cell lysis buffer containing 0.1 mM dithiothreitol and proteinase inhibitor cocktail. Protein concentration was detected using a Bio-Rad DC protein determination kit. Western blot analysis was then performed according to previously described methods (Sun et al., 2011). Immunoblots were developed using an ECL kit. Band intensities were analyzed using the Gel Pro software (Media Cybernetics, Rockville, MD, United States).

### Electrophoretic Mobility Shift Assay

Electrophoretic mobility shift assay (EMSA) was performed according to a commercial kit as previously reported (Meng et al., 2014). Briefly, nuclear protein was extracted from the cerebral cortex for EMSA using a nuclear extraction kit. The protein concentrations were detected using the BCA method. A biotin-labeled, double-stranded DNA oligonucleotide with the NF-κB sequence was purchased from Beyotime Institute of Biotechnology (Jiangsu, China). The sequences of the double strand oligonucleotides were as follows: 5′-ACTGAGGGTGACTCAGCAAAATC-3′ and 3′-TGACTCCCACTGAGTCGTTTTAG-5′ was used for gel shift assays. The nuclear protein was incubated with a biotin-labeled NF-κB probe for 30 min at room temperature in a binding buffer containing 50 μg/mL poly (dI-dC), 5 mM MgCl_2_, 2.5% glycerol, and 0.05% NP-40. Subsequently, the DNA-protein complex was separated by a 6% polyacrylamide gel at 100 V for 1 h. The separated complexes were transferred onto a nylon membrane at 380 mA for 30 min and followed by crosslinked to the nylon membrane. Finally, the membrane was visualized by chemiluminescence using UV light.

### siRNA Assay

siRNA for A20 was obtained from Santa Cruz Biotechnology along with control siRNA and the siRNA transfection reagent according to previous report (Luo et al., 2017). Briefly, 100 nM siRNA was transfected with endothelial cells for 7 h. Then, the endothelial cells were moved to complete medium and incubated for another 24 h. After that, cells were treated with GF1 (16 μM) for 24 h and then exposed to 70 μg/ml of ox-LDL for an additional 24 h. Subsequently, cells were harvested for western blot analysis.

### Molecular Docking

To explore the potential interacting mode of GF1 with the A20 protein (PDB code: 5LRX), a molecular modeling study was performed using SYBYL (X-1.1) according to a previous study (Dai et al., 2015).

### Statistical Analysis

Data are expressed as mean ± SD. The differences between two groups were assessed using a Student’s *t*-test. Data of multiple comparisons were compared using one-way analysis of variance (ANOVA) followed by Tukey’s *post hoc* test. Statistical significance was defined as *p* < 0.05. The calculations were performed using SPSS 19.0 statistical software.

## Results

### GF1 Decreased Atherosclerotic Plaque Development in ApoE-/- Mice

**FIGURE 1:**
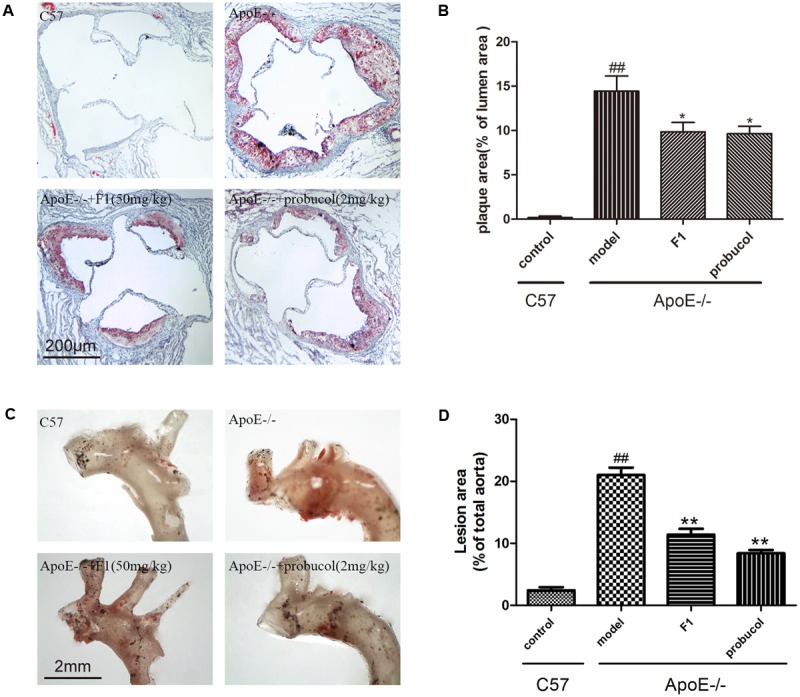
Ginsenoside F1 reduced atherosclerotic plaque development in ApoE-/- mice. All mice were fed HFD. ApoE-/- mice were treated with GF1 (50 mg/kg/day, i.g.), probucol (2 mg/kg, i.g.), or its vehicle (as model group) for 8 weeks; C57 mice were used as the control group. **(A)** Representative light photomicrographs of oil red O-stained sections from the aortic root. **(B)** Statistical analysis of the oil red O-stained sections. **(C)** Representative images of Sudan IV-stained *en face* of the proximal aorta. **(D)** Statistical analysis of aortic AS expressed as a fraction of the total aortic area. Values (*n* = 10 per group) are expressed as the mean ± SD. Values (*n* = 10 per group) are expressed as the mean ± SD. ^##^*p* < 0.01, vs. C57 control group; ^∗^*p* < 0.05, ^∗∗^*p* < 0.01 vs. the vehicle-treated ApoE-/- model group.

Lipid accumulation in aortic arch is a golden method for AS determination. Based on our previously established ApoE-/- atherosclerotic mouse model (Sun et al., 2013), we first measured the effects of GF1 on plaque formation in aortic root. As shown in **Figures 1A,B**, GF1 treated mice significantly reduced the lesion size compared with model group mice. Moreover, we evaluated lesion area by en face analysis and results indicated that GF1 could remarkably relieve Sudan IV-positive area (**Figures 1C,D**), which was consistent with our previous report (Luo et al., 2015b).

### GF1 Ameliorated Inflammatory Responses in HFD-induced ApoE-/- Mice

**FIGURE 2:**
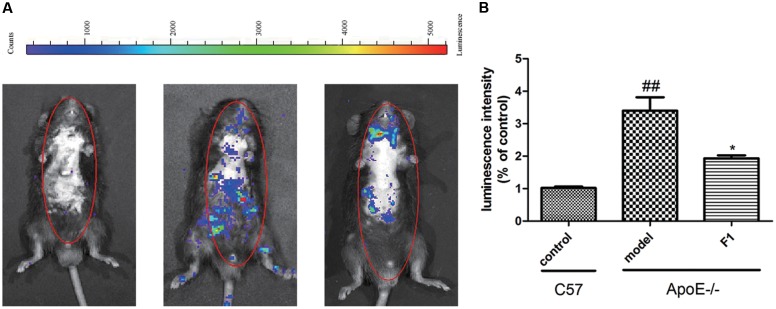
Ginsenoside F1 ameliorated inflammatory responses in HFD-induced ApoE-/- mice. All mice were fed HFD. ApoE-/- mice were treated with GF1 (50 mg/kg/day, i.g.), probucol (2 mg/kg/day, i.g.), or its vehicle (as model group) for 8 weeks; C57 mice were used as the control group. **(A)** Representative photomicrographs of MPO distribution in the whole body. **(B)** Statistical analysis of MPO-positive average luminescence intensity. Values (*n* = 10 per group) are expressed as the mean ± SD. ^##^*p* < 0.01, vs. C57 control group; ^∗^*p* < 0.05 vs. the vehicle-treated ApoE-/- model group.

Atherosclerosis is a chronic inflammatory disease. MPO, as an independent inflammatory marker, promotes atherogenesis and neointima formation in mice (Tiyerili et al., 2016). To investigate the effects of GF1 on inflammatory factors, we examined the MPO expression in ApoE-/- mice. As indicated by **Figure 2**, fluorescence intensity of whole body in mice was significantly higher in model group than control group. Treatment with GF1 sharply normalized the inflammatory severity, which suggested that GF1 could reduce MPO distribution in Apoe-/- mice.

### GF1 Decreased LOX-1, TLR4, and NF-κB Expression at the Aortic Valve in ApoE-/- Mice

**FIGURE 3:**
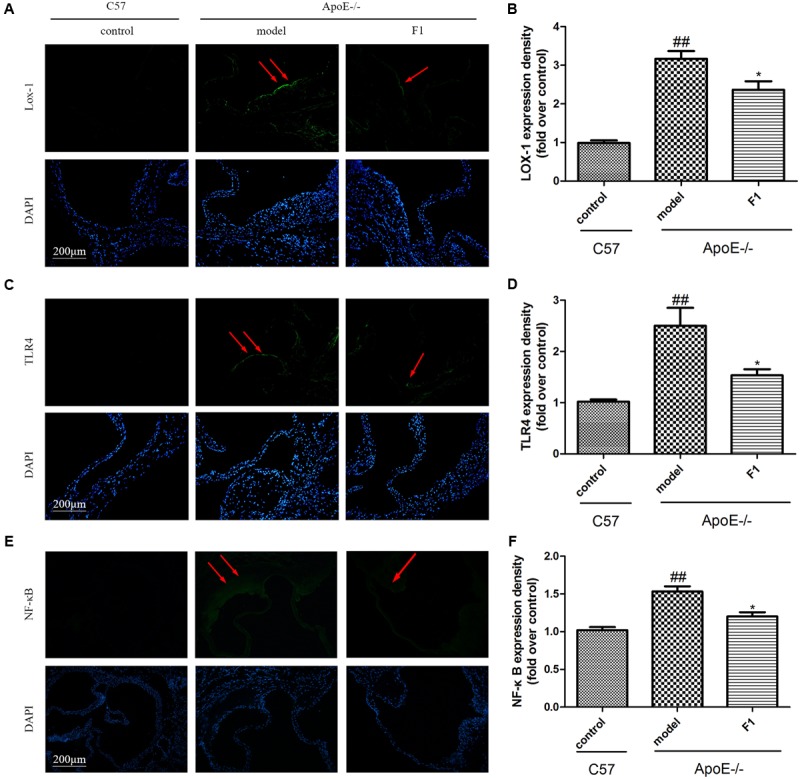
Ginsenoside F1 decreased LOX-1 and TLR4 expression at the aortic valve in ApoE-/- mice. All mice were fed HFD. ApoE-/- mice were treated with GF1 (50 mg/kg/day, i.g.), probucol (2 mg/kg/day, i.g.), or its vehicle (as model group) for 8 weeks; C57 mice were used as the control group. **(A)** Representative images of LOX-1 expression under immunofluorescent staining. **(B)** Quantitative analysis of LOX-1 expression in aortic valve. **(C)** Representative images of TLR4 expression under immunofluorescent staining. **(D)** Quantitative analysis of TLR4 expression in aortic valve. **(E)** Representative images of NF-κB expression under immunofluorescent staining. **(F)** Quantitative analysis of NF-κB expression in aortic valve. Values (*n* = 10 per group) are expressed as the mean ± SD. ^##^*p* < 0.01, vs. C57 control group; ^∗^*p* < 0.05 vs. the vehicle-treated ApoE-/- model group.

LOX-1 and TLR4 are 2 atherosclerotic cell receptors (Zhao et al., 2014), and their high expression suggests the susceptibility of AS. As shown in **Figures 3A–D**, compared with the model group, GF1 treatment group obviously alleviated LOX-1 and TLR4 expression at the aortic valve in ApoE-/- mice. Importantly, GF1 could visibly decreased NF-κB expression **Figures 3E,F**.

### GF1 Prevented ox-LDL-Induced HUVECs Inflammatory Injury

**FIGURE 4:**
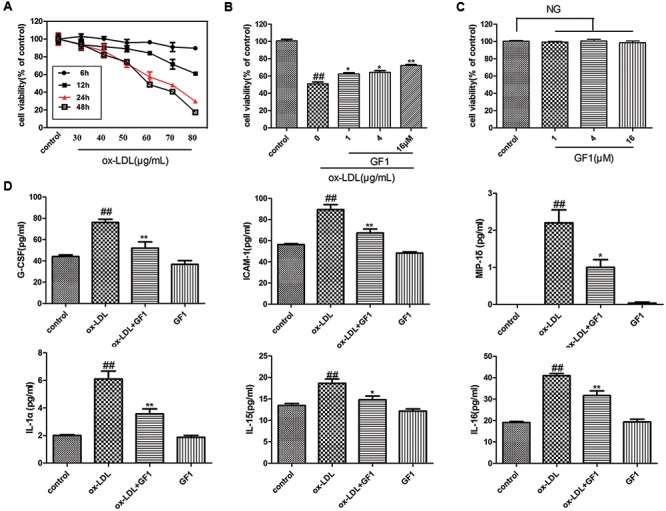
Ginsenoside F1 prevented ox-LDL-induced HUVECs inflammatory injury. **(A)** Effects of different concentration and time of ox-LDL on HUVECs viability. **(B)** Effects of GF1 on ox-LDL-induced HUVECs viability. HUVECs were pretreated with the indicated doses (1, 4, and 16 μM) of GF1 for 24 h before exposure to 70 μg/ml ox-LDL for another 24 h. Cell viability was measured by the MTT assay. **(C)** Effects of GF1 on the viability of HUVECs. Cells were incubated with the indicated doses (1, 4, and 16 μM) of GF1 for 24 h. Cell viability was measured by the MTT assay. **(D)** HUVECs were pretreated with GF1 (16 μM) for 24 h and ox-LDL incubation for 24 h. Proteins of the supernatant related to the inflammatory factors were analyzed using human inflammation antibody array. All of the results are expressed as the mean ± SD of three independent experiments. ^##^*p* < 0.01, vs. control group; ^∗^*p* < 0.05, ^∗∗^*p* < 0.01 vs. ox-LDL group, NG means no significance.

To further investigate the anti-atherosclerotic effects of GF1, we explored the protective effects on ox-LDL-induced endothelial cell injury (**Figure 4A**). As **Figure 4B** shown, GF1 could observably increase cell viability in a dose dependent manner and indicated non-toxic side effects (**Figure 4C**). Meanwhile, gene microarray results demonstrated that GF1 significantly mitigated ox-LDL-induced G-CSF, ICAM-1, MIP-1σ, IL-1α, IL-15, and IL-16 high expression level (**Figure 4D**).

### GF1 Mitigated the Adhesion of THP-1 Cells to HUVECs Stimulated by ox-LDL

**FIGURE 5:**
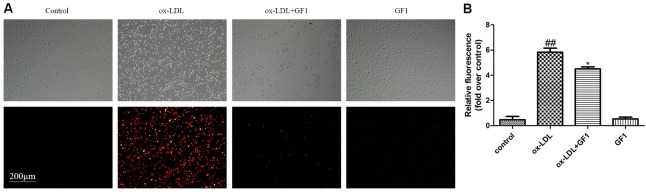
Ginsenoside F1 mitigated the adhesion of THP-1 cells to HUVECs stimulated by ox-LDL. HUVECs were pre-treated with GF1 (16 μM), followed by treatment with ox-LDL for another 24 h. **(A)** Representative images of CM-diL labeled THP-1 cells adherent to HUVECs. **(B)** The quantitative analysis of relative fluorescence of CM-diL labeled THP-1 cells adherent to HUVECs. All of the results are expressed as the mean ± SD of three independent experiments. ^##^*p* < 0.01, vs. control group; ^∗^*p* < 0.05, ^∗∗^*p* < 0.01 vs. ox-LDL group.

Monocytes adhesion is an important feature in early AS progress. As indicated in **Figure 5**, ox-LDL-injured endothelial cell adhered more monocytes compared with control group. Nevertheless, GF1 apparently reduced the adhered monocytes.

### GF1 Suppressed LOX-1, TLR4, and Blocked NF-κB Nuclear Translocation in ox-LDL-Treated HUVECs

**FIGURE 6:**
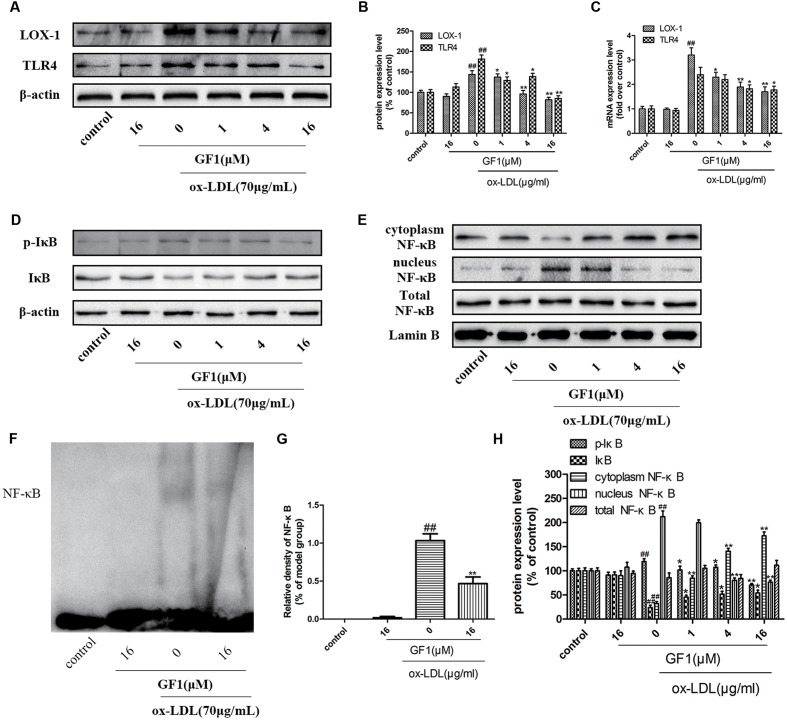
Ginsenoside F1 suppressed LOX-1, TLR4, and blocked NF-κB nuclear translocation in ox-LDL-treated HUVECs. HUVECs were pre-treated with various concentrations of GF1, followed by treatment with ox-LDL for another 24 h. **(A)** Protein expression levels of LOX-1 and TLR4 were measured via western blotting. **(B)** Densitometric analysis was used to quantify the levels of LOX-1 and TLR4. **(C)** Gene expression levels of LOX-1 and TLR4 were measured via real-time PCR. **(D,E)** Protein expression levels of p-IκB, IκB, cytoplasm NF-κB, nucleus NF-Kb, total NF-κB, β-actin and Lamin B were measured via western blotting. **(F)** Representative image of NF-κB activation was evaluated using electrophoretic mobility shift assay. **(G)** Densitometric analysis of NF-κB activation. **(H)** Densitometric analysis was used to quantify the levels of p-IκB, IκB, cytoplasm NF-κB, nucleus NF-κB, and total NF-κB. All of the results are expressed as the mean ± SD of three independent experiments. ^##^*p* < 0.01, vs. control group; ^∗^*p* < 0.05, ^∗∗^*p* < 0.01 vs. ox-LDL group.

LOX-1 is the ox-LDL special receptor, and TLR4 is the major transmembrane receptor mediated inflammatory response. NF-κB is a key downstream regulatory protein for inflammatory factors (Zhao et al., 2014). Our data demonstrated that GF1 could significantly alleviate LOX-1 and TLR4 proteins and mRNA expression (**Figures 6A–C**). Importantly, our results indicated that ox-LDL markedly increased IκB phosphorylation (**Figures 6D–H**), nuclear NF-κB expression level and reduced cytoplasm NF-κB expression level (**Figures 6E–H**), which implied ox-LDL activated NF-κB nuclear translocation. Ox-LDL induced NF-κB nuclear translocation were inhibited by GF1 pretreatment (**Figures 6D–H**). Moreover, the ameliorative effects of GF1 on NF-κB nuclear translocation were confirmed by EMSA assay (**Figures 6F,G**). Taken together, these data suggested that GF1 exerted its anti-inflammatory effects through activating NF-κB pathway.

### GF1 Inhibited NF-κB Signaling via A20 Promotion

**FIGURE 7:**
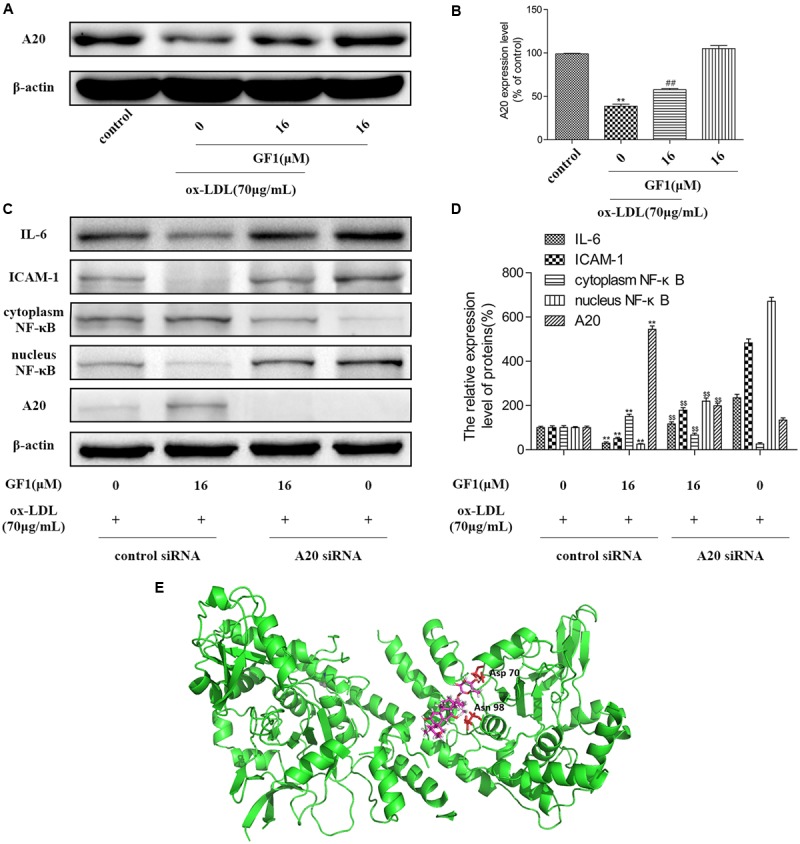
Ginsenoside F1 inhibited NF-κB signaling via A20 promotion. **(A)** HUVECs were pre-treated with various concentrations of GF1, followed by treatment with ox-LDL for another 24 h. Protein expression levels of A20 and β-actin were measured via western blotting. **(B)** Densitometric analysis was used to quantify the levels of A20. **(C)** A20 was knocked down by siRNA, as described in Materials and Methods. At 24 h post-transfection, cells were incubated with GF1 (16 μM) for 12 h and then exposed to ox-LDL for another 24 h. Protein expression levels of A20, IL-6, ICAM-1, cytoplasm NF-κB, nucleus NF-κB and β-actin were measured via western blotting. **(D)** Densitometric analysis was used to quantify the levels of A20, IL-6, ICAM-1, cytoplasm NF-κB, and nucleus NF-κB. **(E)** Docking simulation of GF1 into A20. All of the results are expressed as the mean ± SD of three independent experiments. ^##^*p* < 0.01, vs. control group; ^∗^*p* < 0.05, ^∗∗^*p* < 0.01 vs. ox-LDL group; *p*^$$^< 0.01 vs. ox-LDL+GF1 group.

To further explore the mechanisms of GF1 on NF-κB activation, we measured the A20 expression level. As shown in **Figures 7A,B**, GF1 markedly increased the A20 expression. Next, we evaluated whether the GF1-mediated A20 upregulation was implicated in NF-κB signaling inhibition. Our results showed that the decrease of IL-6, ICAM-1, and increase of NF-κB nuclear translocation mediated by GF1 was abolished by A20 siRNA (**Figures 7C,D**). Moreover, molecular modeling of GF1 indicated that it was bound deeply into the binding cavity of A20 and showed important conventional hydrogen bond interaction with the amino acid residues Asp70 and Asn98 (**Figure 7E**). Altogether, these results suggested that A20 promotion is required for GF1-mediated the NF-κB signaling inhibition in ox-LDL-stimulated HUVECs.

## Discussion

The Ross’s theory indicated that AS is an oxidative lipids causing inflammatory process (Ross, 1993). In clinical, widely used lipid-lowering agents and antioxidants for AS treatment exert partial efficacy. The anti-inflammatory drugs are still deficient (Charo and Taub, 2011). GF1 exerts high anti-cancer and an anti-apoptosis activities (Lee et al., 2003; Kim et al., 2015), but it is scarce in the natural products, which limits its development and utilization. Recently, several groups have shown the GF1 bioconversion (Kim et al., 2011; Wang Y. et al., 2016), which promote the research on its pharmacological activity. With these issues, we investigated the protective effects of GF1 against endothelial cell injury caused AS.

Endothelial cell apoptosis accelerates inflammatory response and promotes macrophages internalize the lipids, which leads to plaque formation (Weber and Noels, 2011). In HFD-induced ApoE-/- mice, GF1 could significantly reduce plaque formation, which was in accordance with our previous results (Qin et al., 2015). We further explored the anti-inflammatory effects, and our results demonstrated that GF1 remarkably reduced MPO expression in atherosclerotic mice. Taken together, these results indicated that GF1 exerts its anti-atherosclerosis effects may be through its anti-inflammatory effects.

Furthermore, we explored the protective effects of GF1 against HUVECs injury. In recent years, ox-LDL is a key factor in the development of AS. Ox-LDL could target its receptor LOX-1, which promotes endothelial cell apoptosis and accelerates AS deterioration (Dominguez et al., 2008). Based on our previous model, we found that GF1 could ameliorated ox-LDL-induced endothelial cell injury in a non-toxic and dose-dependent manner. Meanwhile, GF1 reduced LOX-1 and TLR4 expression, which demonstrated that GF1 ameliorated AS by inhibiting ox-LDL-induced endothelial cell injury. The same results were confirmed in aortic valve in ApoE-/- mice, GF1decreased LOX-1 and TLR4 expression level in atherosclerotic lesion area.

Inflammatory factors secreted by damaged endothelial cells induce monocytes adhesion to endothelial cells, which play a crucial role in the pathogenesis of AS (Bjorkbacka et al., 2013). Next, we investigated the effects of GF1 on ox-LDL-induced endothelial inflammatory response. Based on our protein microarray results, we selected several highly expressed and AS related genes for PCR analysis. Our data showed that GF1 treatment could significantly inhibit inflammatory factors G-CSF, ICAM-1, MIP-1δ, IL-1α, IL-15, IL-16 expression levels and reduce monocytes adherent to the injured endothelial cells.

Nuclear transcription factor NF-κB, a famous regulator, could modulate functional genes expression including inflammatory and adhesion molecules. We have investigated whether NF-κB activity is involved in the protective mechanism of GF1 on ox-LDL-induced endothelial cell injury. As expected, GF1 significantly inhibited NF-κB-luciferase transcription activity in endothelial cells and NF-κB expression in atherosclerotic lesion area. A20, as an inflammatory regulatory protein, has been reported involved in inhibiting NF-κB signaling (Beyaert et al., 2000; Li et al., 2016). Firstly, our data indicated that ox-LDL considerably suppressed the A20 expression, which was in line with the previous study (Heermeier et al., 2001). Ox-LDL may modulate A20 expression through it receptors. Importantly, our data demonstrated that GF1 significantly increased A20 expression level. Expectedly, A20 siRNA markedly abrogated GF1-modulated NF-κB signaling inhibition. It is proposed that GF1 exerts NF-κB activity downregulation by inhibiting IκB phosphorylation via A20-dependent pathway in endothelial cells, which subsequently attenuates atherogenesis.

## Conclusion

**FIGURE 8:**
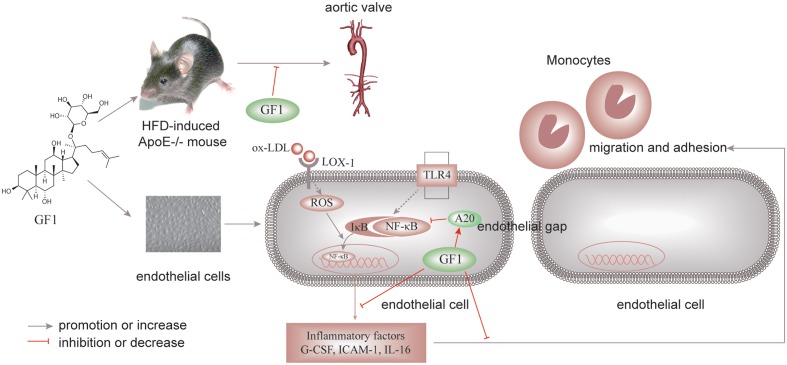
Schematic of GF1 mechanism of preventing ox-LDL-induced inflammatory injuries through activating A20-mediated NF-κB signal pathway.

Ginsenoside F1 could decrease endothelial cell apoptosis, alleviate inflammation in endothelial cells, prevent from the adherence of monocytes to endothelial cells, and postpone the progression of AS via activating A20-mediated NF-κB pathway inhibition (**Figure 8**). Consequently, GF1 presents potential in clinical prophylaxis for AS.

## Author Contributions

MQ and YL performed all the experiments and wrote the manuscript. SL analyzed the data. JS and KY contributed manuscript revision and materials tools. GS and XS designed the study and revised the manuscript.

## Conflict of Interest Statement

The authors declare that the research was conducted in the absence of any commercial or financial relationships that could be construed as a potential conflict of interest. The reviewer OC and handling editor declared their shared affiliation.
